# 

**Published:** 1976

**Authors:** 


Bristol Medico-Chirurgical Journal

(Incorporating the Medical Journal of the South-West)

Established 1883

Vol. 91 (i/ii) JANUARY/APRIL 1976 No. 337/8

### BRISTOL MEDICO-CHIRURGICAL JOURNAL

EDITOR: D. S. REEVES, M.B., M.R.C.Path.

Published Quarterly

Annual Subscription £2 Post Free

### BRISTOL MEDICO-CHIRURGICAL SOCIETY

**President 1975/76:** D. C. Bodenham, Esq.

**President-elect:** Dr. S. J. Silvey.

**Honorary Secretary:** A. J. Webb, Esq., 7 Percival Road, Bristol 8.

**Honorary Treasurer:** Dr. Frank Ross, Bristol Royal Infirmary, Bristol 2.

The Society was founded in 1874. In 1883 publication of the Journal began and has continued quarterly ever since then. During the years 1949 to 1962 it appeared under the title of "The Medical Journal of the South West".

### THE BRISTOL MEDICO-CHIRURGICAL JOURNAL

**Editorial Committee**

Editor Dr. D. S. Reeves, Southmead Hospital, Bristol.

Assistant Editor Dr. A. P. Radford

Members Dr. Rhys Davies.
Dr. M. B. Lennard.
Professor R. C. Wofinden.
Dr. D. W. Wright.
The Hon. Treasurer and Hon. Secretary of the Society.

Secretary Mr. B. P. Jones, The Library, Medical School, University Walk, Bristol BS8 1TD.

### NOTICE TO CONTRIBUTORS

The Journal is especially concerned to provide a place for the recording of medical thought, interests, and practice in and around Bristol. It therefore welcomes articles that, as well as being of immediate interest to members, will document the local and contemporary medical scene for our successors.

Original articles are invited provided that they have not been published elsewhere. References in the text should be by author's name, with year of publication in parenthesis; they should be listed at the end in alphabetical order, the name of each journal or book being given in full—not abbreviated—and the title of the article added. Illustrations should be supplied ready for reproduction. Line drawings should be in Indian ink on firm white paper or board. The author will be informed if it is found necessary to ask him to pay for the making of blocks.

The following contributions will be especially welcome; notes of forthcoming events, meetings held, degrees conferred, staff appointments, honours and public appointments and other local news.

### University of Bristol

### Examinations, Results and Dissertations Approved

FACULTY OF MEDICINE
THE DEGREE OF DOCTOR OF PHILOSOPHY
OCTOBER, 1975
DISSERTATION APPROVED

Victoria Bourn TAGART

FACULTY OF MEDICINE
THE DEGREE OF DOCTOR OF MEDICINE
OCTOBER, 1975
DISSERTATION APPROVED

Richard Maitland AUTY

FINAL EXAMINATION FOR THE DEGREE OF B.D.S.
(SECTION III)
JUNE 1975
PASS

David Vernon BOWLES
Charles Edward COE
Julia Elinor Charlotte DAVIS
Geir Atle KVAMME
Patricia MUSGROVE
Malcolm Leslie PENGELLEY
Carolyn Voirrey SHIMMIN
Chi-Wing WONG

FINAL EXAMINATION FOR THE DEGREE OF B.D.S.
(SECTION III)
DECEMBER 1975
PASS

WITH HONOURS

Peter James BARTER
Stephen John William LISNEY
Paul Lawrence NEWELL

PASS

John Harold ASHTON
Anthony John CHARLES
Peter Leslie DENTON
David EATON
Clive Jonathan ELDER
Guy Anthony FARRANT
Anna FRANCIS
Barry GILLING
David Roger GREEF
Clive Holtom HARRIS
Timothy John HAYWARD
James Michael HOLDER
Ian Carl Anthony JONES
Steven Bernard KNEEBONE
Elizabeth Avril KNOWLES
Gareth Bernard LATHAM
Edward Clement LIZI
Suzanne Margaret MASTERS
Ann Elizabeth MATHIESEN
Martin McNALLY
Rosanne MERRIS
Eileen Mary MORRISON
Elaine Mary MYERSCOUGH
Philip Thomas NUNN
Alan Francis PATTERSON
Julia Faith PICTON
Jeremy Neville PRESTON
Stephen John PROVART
Karen Joanna RANKIN
Philip Lindsay RATCLIFFE
David John REES
Christopher Eric RICKARD
Roger John ROBINSON
Alison Elizabeth RUSSELL
Robert Christopher SANDERSON
Hedley Cyril Jenkin SAUNDERS
Anne Elizabeth SEABRIGHT
Mervyn Reginald Leslie SLUMING
Richard John SMITH
Mary Lesta SUFFIELD
Alan Henry WALKER
Anthony Hayward Robert WELCH
Hon-Kin Andes WU

FACULTY OF MEDICINE
THE DEGREE OF DOCTOR OF PHILOSOPHY
JANUARY, 1976
DISSERTATIONS APPROVED

Peter James BROWN
Jeremy Newton LUCKE
David Gwynfor MORGAN
Iradj POUSTY

FACULTY OF MEDICINE
THE DEGREE OF DOCTOR OF MEDICINE
JANUARY, 1976
DISSERTATIONS APPROVED

Glaciomar MACHADO-OLIVE

Mohsen SHAHMANESH

### NOTICE

The Inaugural dinner of the Bristol Medico-Legal Society was held in the Dragonara Hotel on Friday, 28th February, 1975. Dr. John Apley, the President, introduced the guest of honour Lord Justice Ormrod who spoke wittily of the roles of the doctor and the lawyer in the process of justice. The Society has a membership of 210, and 149 attended the inaugural dinner.

